# Quantification and correction of the scattered X-rays from a megavoltage photon beam to a linac-mounted kilovoltage imaging subsystem

**DOI:** 10.1259/bjro.20190048

**Published:** 2020-12-11

**Authors:** Hiraku Iramina, Mitsuhiro Nakamura, Yuki Miyabe, Nobutaka Mukumoto, Tomohiro Ono, Hideaki Hirashima, Takashi Mizowaki

**Affiliations:** ^1^ Department of Radiation Oncology and Image-applied Therapy, Kyoto University Hospital, Kyoto 606-8507, Japan; ^2^ Division of Medical Physics, Department of Information Technology and Medical Engineering, Human Health Science, Graduate School of Medicine, Kyoto University, Kyoto 606-8507, Japan; ^3^ Department of Radiation Oncology and Image-applied Therapy, Graduate School of Medicine, Kyoto University, Kyoto 606-8507, Japan

## Abstract

**Objective:**

To quantify and correct megavoltage (MV) scattered X-rays (MV-scatter) on an image acquired using a linac-mounted kilovoltage (kV) imaging subsystem.

**Methods and materials:**

A linac-mounted flat-panel detector (FPD) was used to acquire an image containing MV-scatter by activating the FPD only during MV beam irradiation. 6-, 10-, and 15 MV with a flattening-filter (FF; 6X-FF, 10X-FF, 15X-FF), and 6- and 10 MV without an FF (6X-FFF, 10X-FFF) were used. The maps were acquired by changing one of the irradiation parameters while the others remained fixed. The mean pixel values of the MV-scatter were normalized to the 6X-FF reference condition (MV-scatter value). An MV-scatter database was constructed using these values. An MV-scatter correction experiment with one full arc image acquisition and two square field sizes (FSs) was conducted. Measurement- and estimation-based corrections were performed using the database. The image contrast was calculated at each angle.

**Results:**

The MV-scatter increased with a larger FS and dose rate. The MV-scatter value factor varied substantially depending on the FPD position or collimator rotation. The median relative error ranges of the contrast for the image without, and with the measurement- and estimation-based correction were −10.9 to −2.9, and −1.5 to 4.8 and −7.4 to 2.6, respectively, for an FS of 10.0 × 10.0 cm^2^.

**Conclusions:**

The MV-scatter was strongly dependent on the FS, dose rate, and FPD position. The MV-scatter correction improved the image contrast.

**Advances in knowledge:**

The MV-scatters on the TrueBeam linac kV imaging subsystem were quantified with various MV beam parameters, and strongly depended on the fieldsize, dose rate, and flat panel detector position. The MV-scatter correction using the constructed database improved the image quality.

## Introduction

Image-guided radiotherapy (IGRT) systems have been employed extensively over the past two decades.^1,2^ Kilovoltage (kV) and megavoltage (MV) X-rays are used as radiation-based techniques for IGRT, whereas magnetic resonance imaging, ultrasound, optical imaging, and surface imaging are used as non-radiation-based techniques. These methods may be used either alone or in combination.

A modern medical linear accelerator (linac) may have one or two kV imaging subsystems that arelocated perpendicular to or at ±135° interval with respect to the therapeutic MV beam. Independent kV imaging subsystems with two or four kV sources and detectors have also been used.kV imaging subsystems are used for tumor localization before therapeutic MV beam irradiation. In recent years, concurrent kV imaging using linac-mounted kV imaging subsystems has garnered attention for use in real-time three-dimensional (3D) IGRT during MV beam irradiation. Such systems include real-time tumor-tracking radiotherapy (RTRT),^3,4^ dynamic tumor tracking with a gimbaled head on Vero4DRT (Mitsubishi Heavy Industries, Ltd., Hiroshima, Japan, and BrainLAB AG, Feldkirchen, Germany),^5^ kilovoltage intrafractional monitoring (KIM),^6^ combined MV and kV imaging,^7^ combined optical and sparse monoscopic imaging with kV X-rays,^8^ and in-treatment cone-beam computed tomography (CBCT) imaging.^9^ A prerequisite for all of these techniques is concurrent kV imaging during MV beam irradiation, whereby the scattered X-rays of the MV beam from scatterers (MV-scatters) are incident on the kV detectors. MV-scatter may degrade the image contrast or visibility of not only concurrent kV images, but also CBCT images, without depending on the kV imaging parameters. For example, the MV-scatter on a concurrent kV image reduces the accuracy of the marker or target detection (Supplementary Material 1). Therefore, all of the above-mentioned real-time 3D IGRT techniques would require an MV-scatter correction method.

The MV-scatter maps acquired for various MV beam parameters should be investigated to establish an extensive MV-scatter correction method for concurrent kV projections, as these projections are necessary for both concurrent CBCT imaging and real-time 3D IGRT techniques using linac-mounted kV imaging subsystems. However, few such studies have been conducted to date. Iramina *et al* investigated the characteristics of MV-scatter on Vero4DRT with two orthogonal kV imaging subsystems.^10^ Although Luo *et al* reported a lower image contrast with a larger field size (FS), MV energy, and dose rate, as well as a closer flat-panel detector (FPD) position, the variations in each parameter tested in the study were limited and the dependencies were not determined.^11^ Wallace *et al* investigated the effect of MV-scatter to optimize the parameters for their KIM method, but no information on the MV-scatter map itself was made available.^12^

To the best of the authors' knowledge, no study has yet investigated the dependencies of the parameters on the MV-scatter for a linac with one kV imaging subsystem in detail, including the use of flattening-filter-free (FFF) beams. The purpose of the present study was to quantify the basic physical characteristics of the MV-scatter itself by acquiring an MV-scatter map using a TrueBeam STx linac (Varian Medical Systems, Palo Alto, CA) with various parameters and to construct an MV-scatter database for MV-scatter correction. Different FSs, phantom sizes and densities, dose rates, gantry and collimator angles, and FPD positions from the isocenter were evaluated. Moreover, MV-scatter maps were acquired using not only flattening-filter (FF) but also FFF beams, as the latter can achieve a high dose rate. An MV-scatter correction experiment was also performed, in which an MV-scatter map was subtracted from a kV image acquired during MV beam irradiation by using a phantom including a pseudotumor ball.

## Methods and materials

### MV-scatter map acquisition procedure

The MV-scatter map acquisitions were performed on Developer Mode. A phantom was irradiated with an MV beam. During the MV beam irradiation, the FPD was activated without kV X-ray irradiation to acquire the MV-scatter image using the kV imaging subsystem, conducted with the “DynamicGain-DF” option. A total of 10 images were acquired at nearly 10 MU and without image correction, and the acquisition time of each image was fixed. The first (dark-field) image was acquired with neither MV nor kV irradiation, and thus, displayed the background signal. The dark-field image was subtracted from images 2 to 10. Thereafter, the subtracted images were averaged, yielding the MV-scatter map.

### Parameter variation

**Figure 1.**
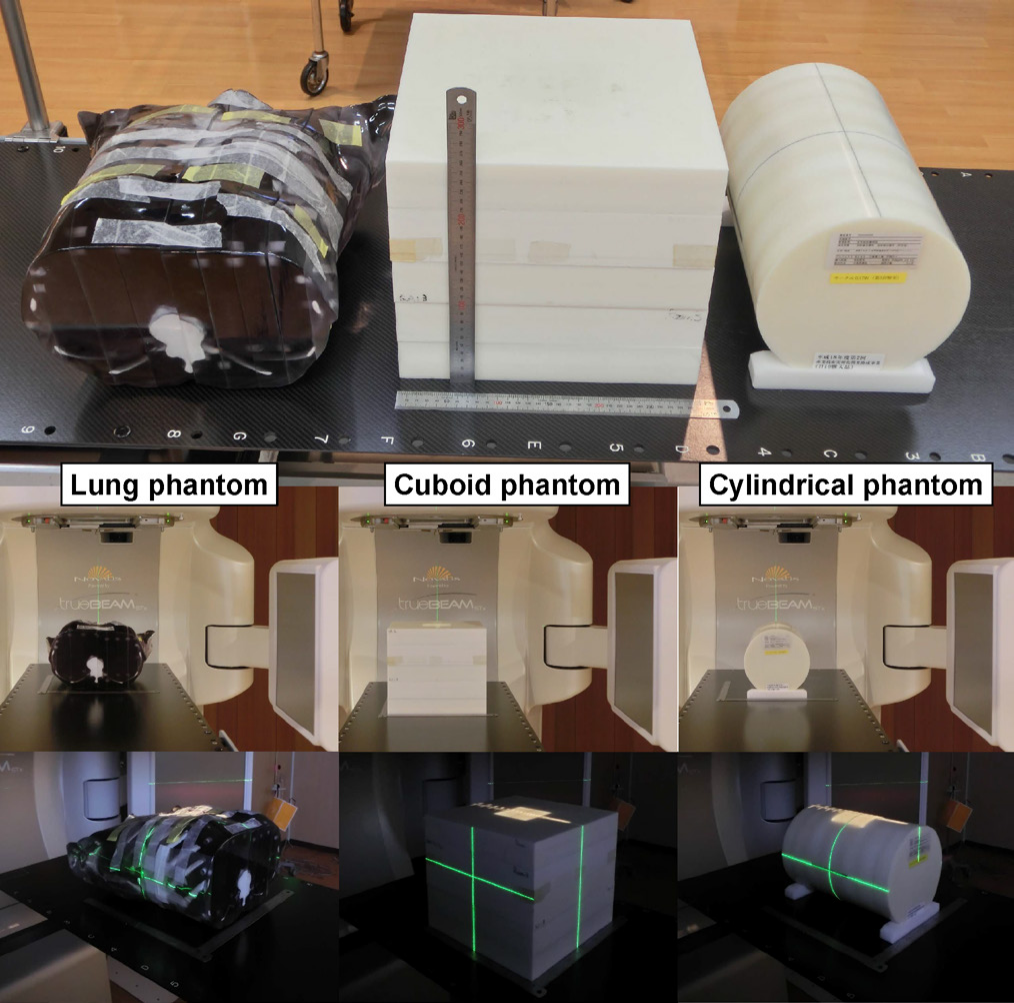
Images of phantoms used in this study: (left) 3D-printed thoracic phantom (Lung phantom), (middle) water-equivalent cuboid phantom (Cuboid phantom), and (right) water-equivalent cylindrical phantom (Cylindrical phantom). The length of the rulers in the middle of the top row is 30 cm. 3D, three-dimensional.

In this study, 6, 10, and 15 MV FF beams (6X-FF, 10X-FF, and 15X-FF, respectively) and 6 and 10 MV FFF beams (6X-FFF and 10X-FFF, respectively) were used. The reference condition was an FS of 10.0 × 10.0 cm^2^, a dose rate of 400 MU/min, gantry and collimator angles of 0°, and an FPD position of 70 cm from the isocenter. The reference scatterer was the water-equivalent cuboid phantom (Taisei Medical, Inc., Osaka, Japan; physical density:~1 g/cm^3^; 30.0 × 30.0 × 26.0 cm^3^; “Cuboid phantom”) set up at a source-to-surface distance (SSD) of 90 cm and a source-to-axis distance of 100 cm. The parameter dependencies were demonstrated by varying one parameter at a time while maintaining the others fixed. The following parameters were tested: FS, dose rate, gantry and collimator angles, and FPD position from the isocenter (Table 1). The 3D-printed anthropomorphic thoracic phantom (Yasojima Proceed, Co., Ltd., Hyogo, Japan; 0, ~ 1, and ~2 g/cm^3^ for lung, soft tissue, and bone regions, respectively; “Lung phantom”) and the water-equivalent cylindrical phantom (Taisei Medical, Inc., Osaka, Japan;~1 g/cm^3^; 20.0φ × 30.0 cm^3^;“Cylindrical phantom”) were used for further considerations of the FS and gantry angle dependencies, respectively (Figure 1). These phantoms were set up at an SSD of 90 cm. After demonstrating all parameter variations through measurements, an MV-scatter database was constructed using the results of 6X-FF.

**Table 1.** Reference condition for the MV-scatter map, variable parameters, and scatterers used in this study

Parameter	Description	Scatterer
Reference condition for MV-scatter map acquisition	Field size: 10.0 × 10.0 cm^2^, dose rate: 400 MU/min, gantry and collimator angles: 0°, flat-panel detector (FPD) position from isocenter: 70 cm	Cuboid
Field size [cm^2^]	2.5 × 2.5, 5.0 × 5.0, 7.5 × 7.5, 10.0 × 10.0, 12.5 × 12.5, 15.0 × 15.0, 17.5 × 17.5, 20.0 × 20.0, 22.5 × 22.5, 25.0 × 25.0, 27.5 × 27.5, 30.0 × 30.0	Cuboid and Lung
Dose rate [MU/min]	6, 10, and 15 MV beam with flattening-filter (FF): 20, 60, 100, 200, 300, 400, 500, 600; 6 MV beam without FF: 400, 600, 800, 1,000, 1,200, 1,400; 10 MV beam without FF: 400, 800, 1,200, 1,600, 2,000, 2,400	Cuboid
Gantry angle [°]	0, 15, 30, 45, 60, 75, 90, 105, 120, 135, 150, 165, 180, 195, 210, 225, 240, 255, 270, 285, 300, 315, 330, 345	Cuboid and Cylindrical
Collimator angle^a^ [°]	0, 15, 30, 45, 60, 75, 90, 105, 120, 135, 150, 165, 175, 185, 195, 210, 225, 240, 255, 270, 285, 300, 315, 330, 345	Cuboid
FPD position from the isocenter [cm]	40, 50, 60, 70, 80	Cuboid

MV, megavoltage.

a Due to mechanical limitations, the movement range of the collimator angle ranged from 185°/195° to 195°/185° counterclockwise/clockwise.

### MV-scatter correction experiment

#### Phantom setup and experimental procedure

A QUASAR phantom (Modus Medical Device, Inc., London, Canada) was used for the MV-scatter correction experiment. A wooden rod (0.4 g/cm^3^) with a 30 mm-diameter spherical pseudo-tumor ball (target ball, 1.05 g/cm^3^) located at the center of the rod was surrounded by a uniform acrylic phantom. The target ball center was positioned to coincide with the isocenter and the longitudinal axis of the rod was parallel to the superior–inferior direction.

Developer Mode was used. In the experiment, three image types were obtained: (#1) kV images without MV beam irradiation for reference (kV only images), (#2) concurrent kV images during MV beam irradiation (MV+kV images), and (#3) images containing MV-scatter only. The kV imaging parameters for each image were 125 kV, 60 mA, and 20 ms. The “DynamicGainFluoro” mode was used and the frame rate was 7 fps. The #3 images could be acquired using the same procedure as that of the #2 images. However, the kV collimators were closed during the concurrent imaging so that the FPD collected MV-scatters only. The MV beam parameters were as follows: the MV beam energies were 6X-FF, 10X-FF, 15X-FF, 6X-FFF, and 10X-FFF (dose rate: 400 MU/min) andthe FSs were 5.0 × 5.0 and 10.0 × 10.0 cm^2^for each energy. The collimator angle was 0° and the FPD position was 70 cm. The gantry was fullyrotated during the image acquisitions and the total number of images obtained was 420.Furthermore, the #3 images of the QUASAR and Cuboid phantom under the reference condition (FS: 10.0 × 10.0 cm^2^, dose rate: 400 MU/min, gantry and collimator angles: 0°, and FPD position: 70 cm from the isocenter) with 6X-FF were acquired.

### MV-scatter correction and evaluation

To correct the MV-scatters from the MV +kV images $P_{MV+kV}$, corresponding images containing MV-scatter $P_{MVSmap}$ needed to be subtracted, and the subtracted images were referred to as the MV-scatter corrected (MVScorr) images $P_{MVScorr}$:

(1) $$P_{MVScorr}=P_{MV+kV}-P_{MVSmap}$$

**Figure 2.**
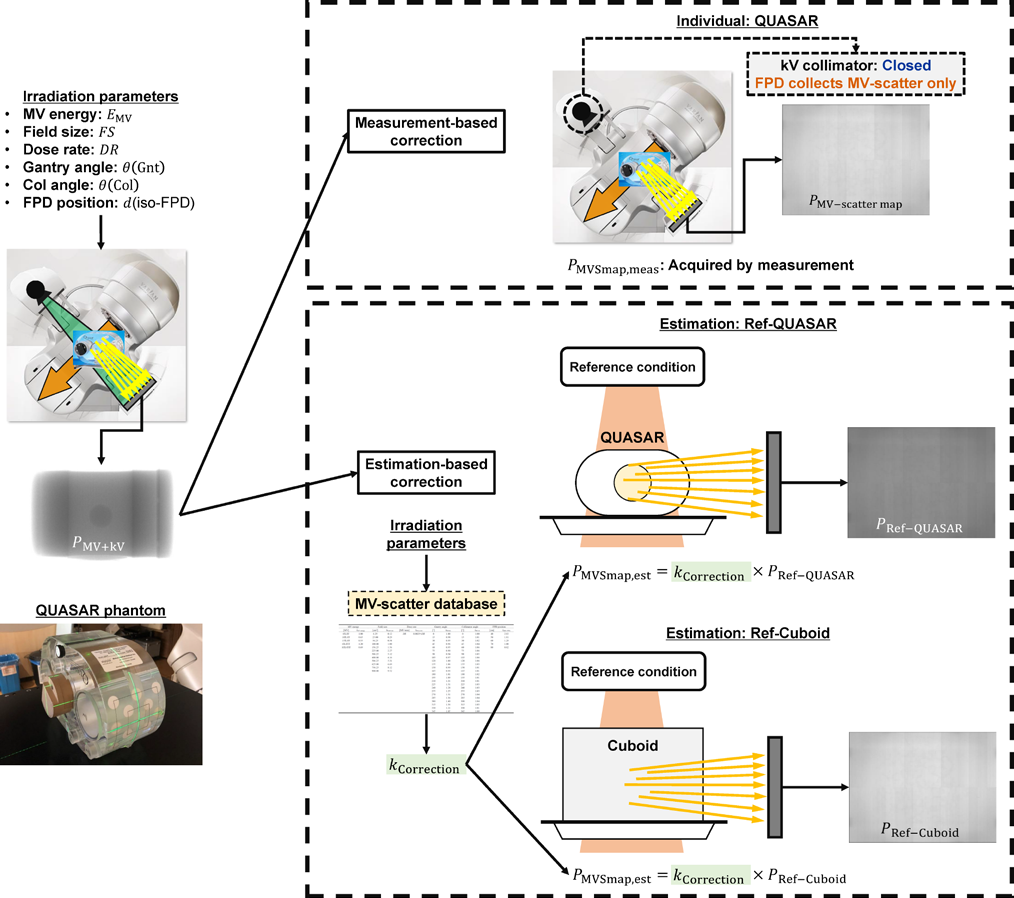
Schematic of MV-scatter correction procedures. MV, megavoltage.

In this study, two MV-scatter correction methods were used: measurement-based and estimation-based (Figure 2). In the measurement-based method, the measured #3 images of each MV beam parameter were used as $P_{MVSmap}$ $\left(P_{MVSmap,meas}\right)$. The correction procedure was referred to as “Individual: QUASAR.” In the estimation-based method, $P_{MVSmap}$ were estimated from one reference image $P_{Ref}$ and various correction factors $\left(k_{Correction}\right)$ obtained from the MV-scatter database and the irradiation parameters:

(2) $$P_{MVSmap,est}=k_{Correction}\times P_{Ref}$$

(3) $$k_{Correction}=k_{MV\;energy}\times k_{Field\;size}\times k_{Dose\;rate}\;\;\times k_{\theta (Gnt)}\times k_{\theta (Col)}\times k_{d(iso-FPD)}$$

where, $k_{MV\;energy},\;k_{Field\;size}$, $k_{Dose\;rate}$, $k_{\theta (Gnt)}$, $k_{\theta (Col)}$, and $k_{d(iso-FDP)}$ are correction factors for the MV beam energy, MV FS, dose rate, gantry angle, collimator angle, and FPD position, respectively. Two images were used for $P_{Ref}$ : the MV-scatter only image of the QUASAR phantom or Cuboid phantom acquired by the reference condition with 6X-FF ($P_{Ref-QUASAR}\;or\;P_{Ref-Cuboid}$, respectively). The correction procedures were referred to as “Estimation: Ref-QUASAR” or “Estimation: Ref-Cuboid,” respectively.

To evaluate the correction, two ROIs (70 × 70 pixels) were set at the center of the target ball (ROI_target_) and the nearby background (ROI_bg_) of the kV only, MV+kV, and MVScorr images. The intensity signals in ROI_target_ and ROI_bg_were averaged, and these averaged signals were referred to as *M*_t_ and *M*_b_, respectively. The image contrast was calculated by the absolute difference between *M*_t_ and *M*_b_ for each image (Supplementary Material 1 Section S4). The relative errors of the MV +kV and MV-scatter corrected images to the kV only image were calculated angle by angle.The MV-scatter correction and image contrast calculation were performed using in-house software developed in MATLAB 2018a (MathWorks, Natick, MA).

## Results

### MV-scatter maps of reference condition

**Figure 3.**
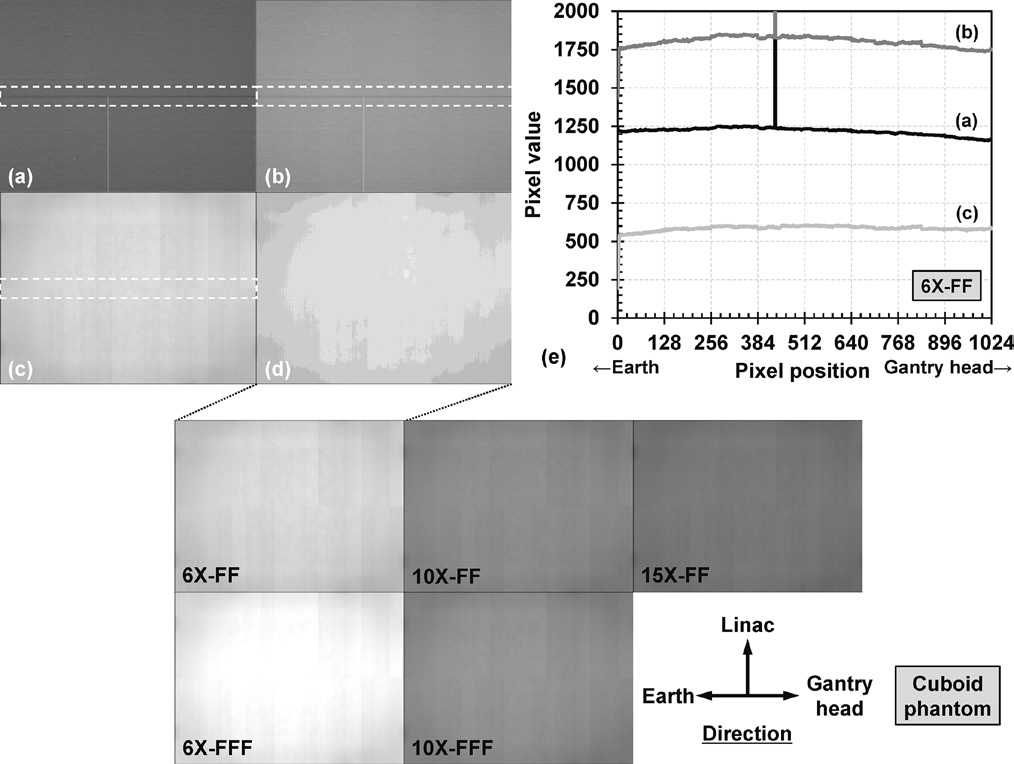
(a) Dark-field image, (b) second image, (c) subtracted image, and (d) averaged image (MV-scatter map) under reference condition of 6 MV photon beam with flattening-filter (6X-FF). The window levels and widths for the pixel value were 1500 and 3000 for (a, b), and 350 and 700 for (c, d), respectively. (e) The pixel value profiles are indicated in the white dashed rectangles in (a–c). The MV-scatter maps of the reference condition acquired by 6X-FF, 10X-FF, 15X-FF, 6 MV photon beam without flattening-filter (6X-FFF), and 10X-FFF are presented in the center. The window level and width for the pixel value were 350 and 700, respectively. FFF, flattening-filter-free; MV, megavoltage.

The dark-field image, second image, subtracted image, and averaged image (*i.e* the MV-scatter map) obtained under the reference condition of 6X-FF are presented in Figure 3. The pixel value distributions for all acquired dark-field images were the same throughout this study. An ROI for the transverse direction (profile ROI_trans_) of 1024 × 100 pixels was defined and the pixel value profiles of the dark-field, second, and subtracted images were subsequently plotted [Figure 3 (e)]. The pixel value profile in the profile ROI_trans _on the MV-scatter map [Figure 3 (d); profile not shown] was almost the same as that on the subtracted image [Figure 3 (c)]. The MV-scatter maps of the reference condition acquired using all MV energies are also shown.

### Dependencies of each parameter

**Figure 4.**
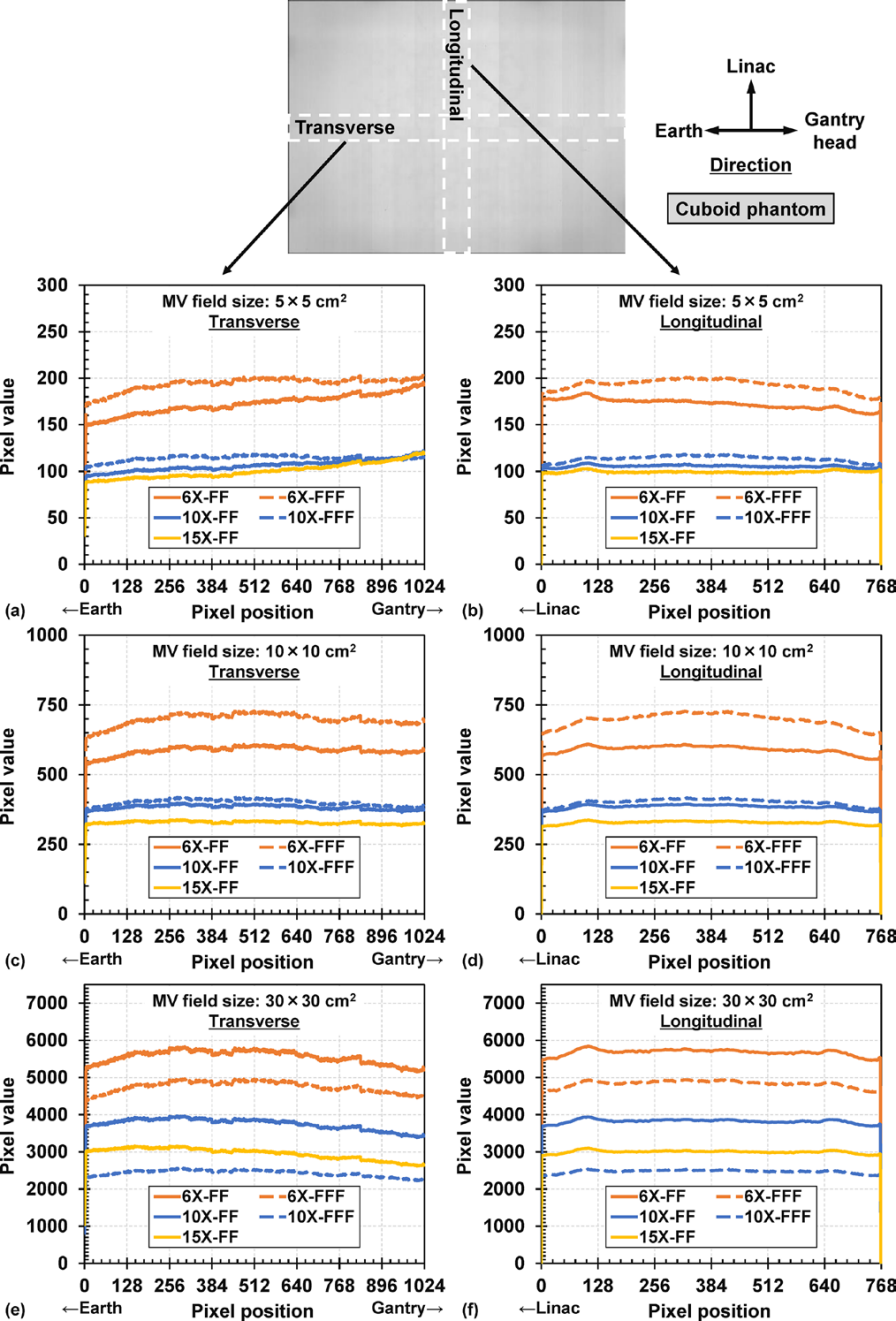
Transverse pixel value profiles of MV-scatter maps acquired with MV FS of (a) 5.0 × 5.0, (c) 10.0 × 10.0, and (e) 30.0 × 30.0 cm^2^. Longitudinal pixel value profiles of MV-scatter maps acquired with MV FS of (b) 5.0 × 5.0, (d) 10.0 × 10.0, and (f) 30.0 × 30.0 cm^2^. FS, field size; MV, megavoltage.

The transverse and longitudinal pixel value profiles on the MV-scatter maps with 5.0 × 5.0, 10.0 × 10.0, and 30.0 × 30.0 cm^2^ are illustrated in Figure 4. The pixel value profiles in both ROIs decreased with higher MV beam energies. The absolute pixel value for the FS of 30.0 × 30.0 cm^2^ was 10-fold larger than that for the FS of 10.0 × 10.0 cm^2^.

**Figure 5.**
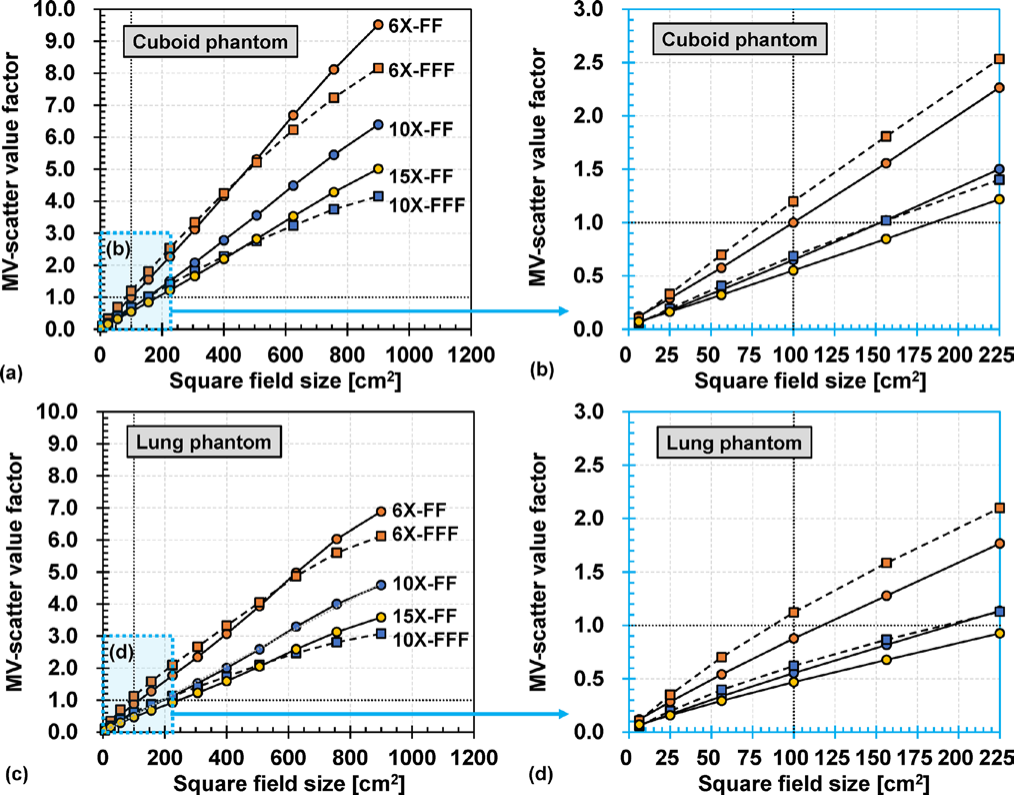
FS dependencies for (a) Cuboid and (c) Lung phantoms with various MV photon beam energies. Results obtained with square FSs ranging from 2.5 × 2.5 to 15.0 × 15.0 cm^2^ for (b) Cuboid phantom and (d) Lung phantom. The intersections of the two dashed lines in (a–d) indicate the MV-scatter value obtained under the reference condition of 6 MV photon beam with flattening-filter (6X-FF). FS, field size; FF, flattening-filter.

The mean pixel values of the MV-scatter were obtained by defining an ROI of 100 × 100 pixels at the center of each MV-scatter map. Thereafter, the mean pixel values were normalized to that of the 6X-FF reference condition (the MV-scatter value). The FS dependencies of the MV-scatter value of the Cuboid phantom are shown in Figure 5 (a) and (b). The MV-scatter value increased with an increasing square FS. Thus, the MV-scatter value of 6X-FF was quadrupled when the square FS was increased from 10.0 × 10.0 to 20.0 × 20.0 cm^2^. The MV-scatter maps of the Lung phantom were also acquired with various square FSs, as shown in Figure 5(c) and (d). The MV-scatter map profile shape for the Lung phantom was the same as that for the Cuboid phantom (Supplementary Material 1 Figure S2). The increase in the MV-scatter value for the Lung phantom was similar to that of the Cuboid phantom, but the increase rate was lower. It was because that the density of the Lung phantom was different from that of the Cuboid phantom. In addition, for the field sizes smaller than certain sizes (such as ≤20.0 × 20.0 cm^2^ for 6X and ≤12.5 × 12.5 cm^2^ for 10X on the Cube phantom, and ≤22.5 × 22.5 cm^2^ for 6X and ≤15.0 × 15.0 cm^2^ for 10X on the Lung phantom), the MV-scatter value factor for the FF beam was smaller than that for the FFF beam. For large field sizes, this trend was inverted due to the convex profile of the FFF beam, such that the fluence around the field edge was less than that at the center of the beam axis.

**Figure 6.**
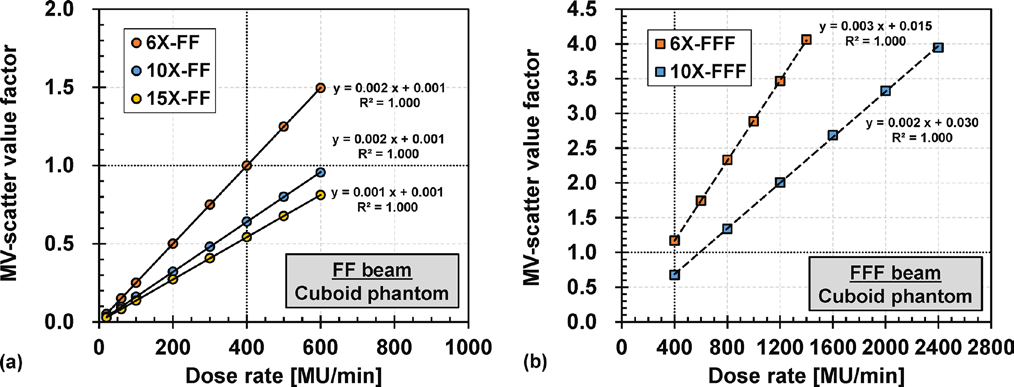
Dose rate dependencies for (a) 6, 10, and 15 MV photon beam with flattening-filter (6X-FF, 10X-FF, and 15X-FF) and (b) 6 and 10 MV photon beam without flattening-filter (6X-FFF and 10X-FFF). The intersections of the two dashed lines in (a, b) indicate the MV-scatter value obtained under the reference condition of 6X-FF. FF, flattening-filter; MV, megavoltage.

The doserate dependencies of the FF and FFF beams are presented in Figure 6 (a) and (b), respectively. The MV-scatter value increased linearly with increasing dose rates. The Pearson’s coefficient of determination for each MV beam energy was almost 1 and the intercept of the fitted linear line was almost 0.

**Figure 7.**
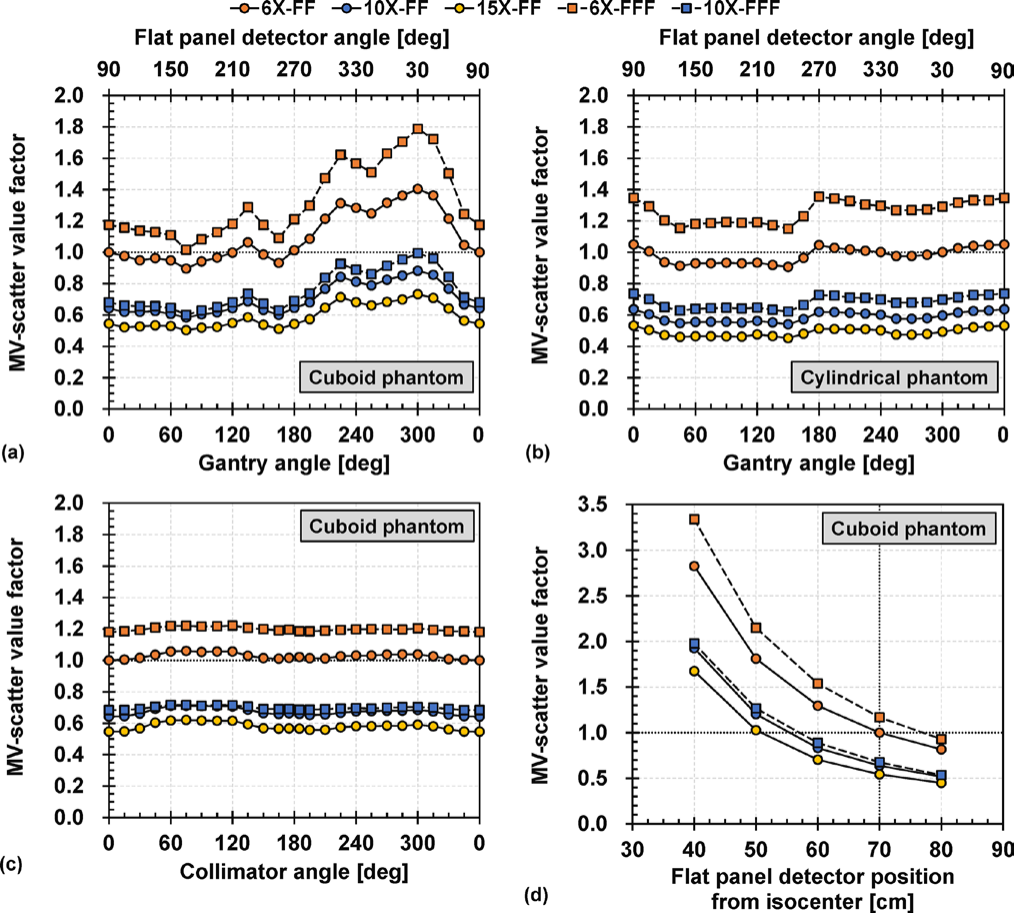
Gantry angle dependencies of (a) Cuboid phantom and (b) Cylindrical phantom with various MV photon beam energies. The dashed line indicates the MV-scatter value obtained under the reference condition of 6 MV photon beam with flattening-filter (6X-FF). (c) Collimator angle dependencies with various MV photon beam energies. The dashed line indicates the MV-scatter value obtained under the reference condition of 6X-FF.(d) FPD position dependencies with various MV photon beam energies. The intersection of the two dashed lines indicates the MV-scatter value obtained under the reference condition of 6X-FF. FF, flattening-filter; FPD, flat panel detector; MV, megavoltage.

The gantry angle dependencies of the Cuboid and Cylindrical phantoms are illustrated in Figure 7 (a) and (b), respectively. For the Cuboid phantom, the MV-scatter value factor varied with the gantry angle, by a maximum of 1.40 for 6X-FF. For the Cylindrical phantom, the largest 6X-FF MV-scatter value factor of 1.05 was obtained at gantry angles of 0° and 90°.

The collimator angle dependencies are shown in Figure 7 (c). A collimator angle of 180° was not possible owing to the mechanical limits; instead, measurements were conducted at 175° and 195°. The 6X-FF, 10X-FF, 15X-FF, 6X-FFF, and 10X-FFF MV-scatter value factors obtained at a collimator angle of 90° were approximately 1.05, 1.10, 1.13, 1.03, and 1.04, respectively, compared to those obtained at a collimator angle of 0°.

The FPD position dependencies are presented in Figure 7 (d). The MV-scatter increased with an increasing proximity of the FPD to the isocenter. The MV-scatter values followed the inverse square law.

An MV-scatter database was constructed using the measurement results of the parameter variations (Table 2). In this case, $k_{MV\;energy}$ was the MV-scatter value factor under the reference condition (Figure 5 (a) or (b)), whereas $k_{Field\;size}$, $k_{Dose\;rate}$, $k_{\theta (Gnt)}$, $k_{\theta (Col)}$, and $k_{d(iso-FPD)}$ were the MV-scatter value factors of 6X-FF from (Figure 5)(a), (Figure 6) (a) and (Figure 7) (a), (c), and (d), respectively. In addition, MV-scatter databases for each MV beam were also constructed (Supplementary Material 1 Table S1-S5). More details were in Supplementary Material 1 Section S5.

**Table 2.** MV-scatter database based on the Cuboid phantom results

MV energy		Field size		Dose rate		Gantry angle		Collimator angle		FPD position	
[MV]	*k*_MV energy_	[cm^2^]	*k*_Field size_	[MU/min]	*k*_Dose rate_	[°]	*k_θ_*_(Gnt)_	[°]	*k_θ_*_(Col)_	[cm]	*k_d_*_(iso-FPD)_
6X-FF 10X-FF 15X-FF 6X-FFF 10X-FFF	1.00 0.65 0.55 1.20 0.69	6.25 25.00 56.25 100.00 156.25 225.00 306.25 400.00 506.25 625.00 756.25 900.00	0.12 0.29 0.58 1.00 1.56 2.27 3.12 4.16 5.31 6.69 8.12 9.52	*DR*	0.0025 × *DR*	0 15 30 45 60 75 90 105 120 135 150 165 180 195 210 225 240 255 270 285 300 315 330 345	1.00 0.98 0.95 0.96 0.95 0.90 0.94 0.97 1.00 1.06 0.99 0.93 1.01 1.09 1.21 1.31 1.28 1.25 1.31 1.36 1.40 1.36 1.21 1.05	0 15 30 45 60 75 90 105 120 135 150 165 180 195 210 225 240 255 270 285 300 315 330 345	1.00 1.01 1.02 1.04 1.06 1.06 1.05 1.06 1.06 1.03 1.01 1.01 1.02 1.01 1.01 1.03 1.03 1.03 1.04 1.04 1.04 1.03 1.01 1.00	40 50 60 70 80	2.83 1.81 1.29 1.00 0.82

FPD, flat-panel detector; MV, megavoltage.

### MV-scatter correction experiment using phantom

**Figure 8.**
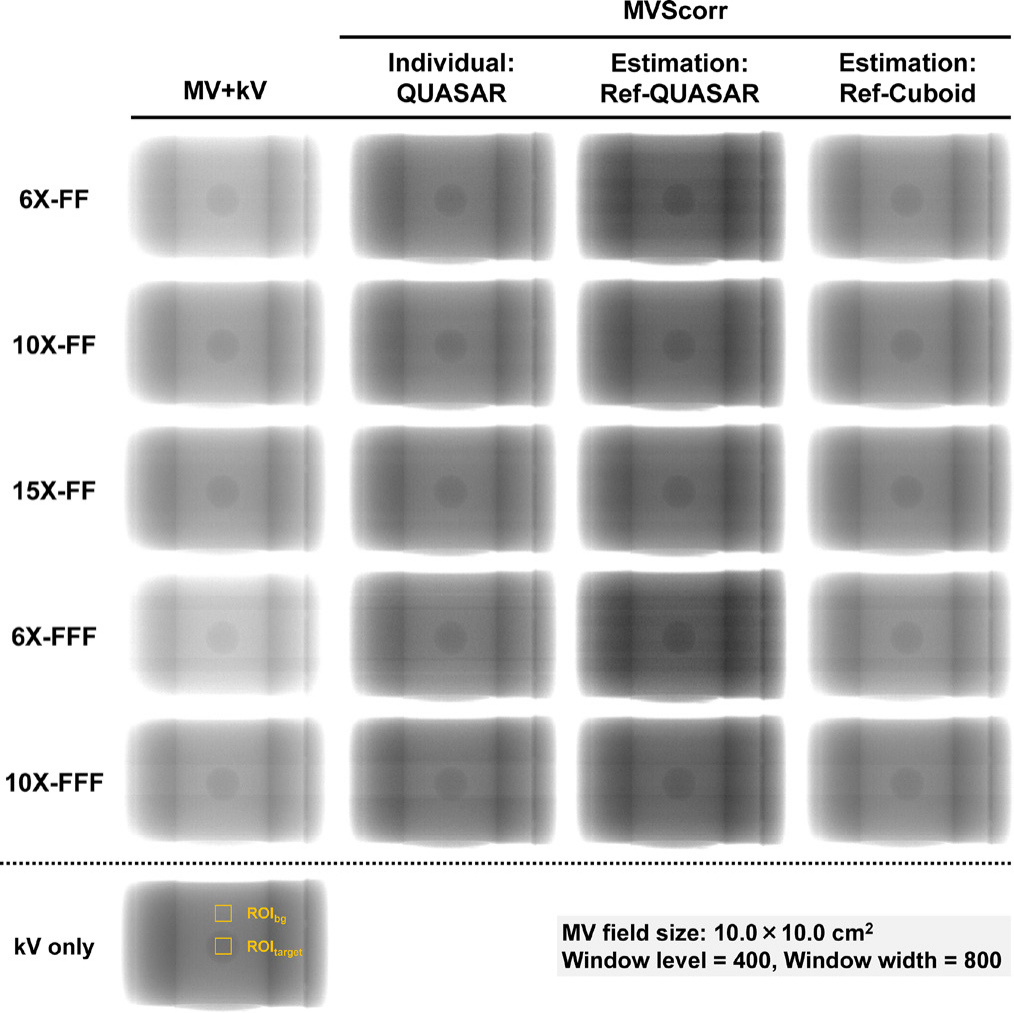
kV images acquired during MV beam irradiation (MV+kV) with FS of 10.0 × 10.0 cm^2^, where MV-scatter corrected images (MVScorr) and kV image without MV beam irradiation are shown. The Individual: QUASAR = MV+kV images were corrected by the MV-scatter map acquired using QUASAR at each gantry angle.The Estimation: Ref-QUASAR = MV-scatter maps were estimated from the database and MV-scatter image of QUASAR phantom acquired by the reference condition (FS: 10.0 × 10.0 cm^2^, dose rate: 400 MU/min, gantry and collimator angles: 0°, and FPD position: 70 cm from isocenter) with 6 MV photon beam with flattening-filter (6X-FF). The Estimation: Ref-Cuboid = MV-scatter maps were estimated from the database and MV-scatter image of the Cuboid phantom acquired by the reference condition of 6X-FF.The window level and width were 400 and 800, respectively. FF, flattening-filter; FPD, flat panel detector; MV, megavoltage.

The kV only, MV+kV,and MVScorr images with an FS of 10.0 × 10.0 cm^2^ at gantry angles of 180°are illustrated in Figure 8. From the qualitative view, the MV-scatter was increased with lower MV beam energies. More MV-scatter was incident on the FPD for 6X-FFF beam compared to 6X-FF, supported by Figure 5 (b). Compared to the MV+kV images, the MV-scatter was eliminated and the MVScorr images were comparable with the kV only image.

**Figure 9.**
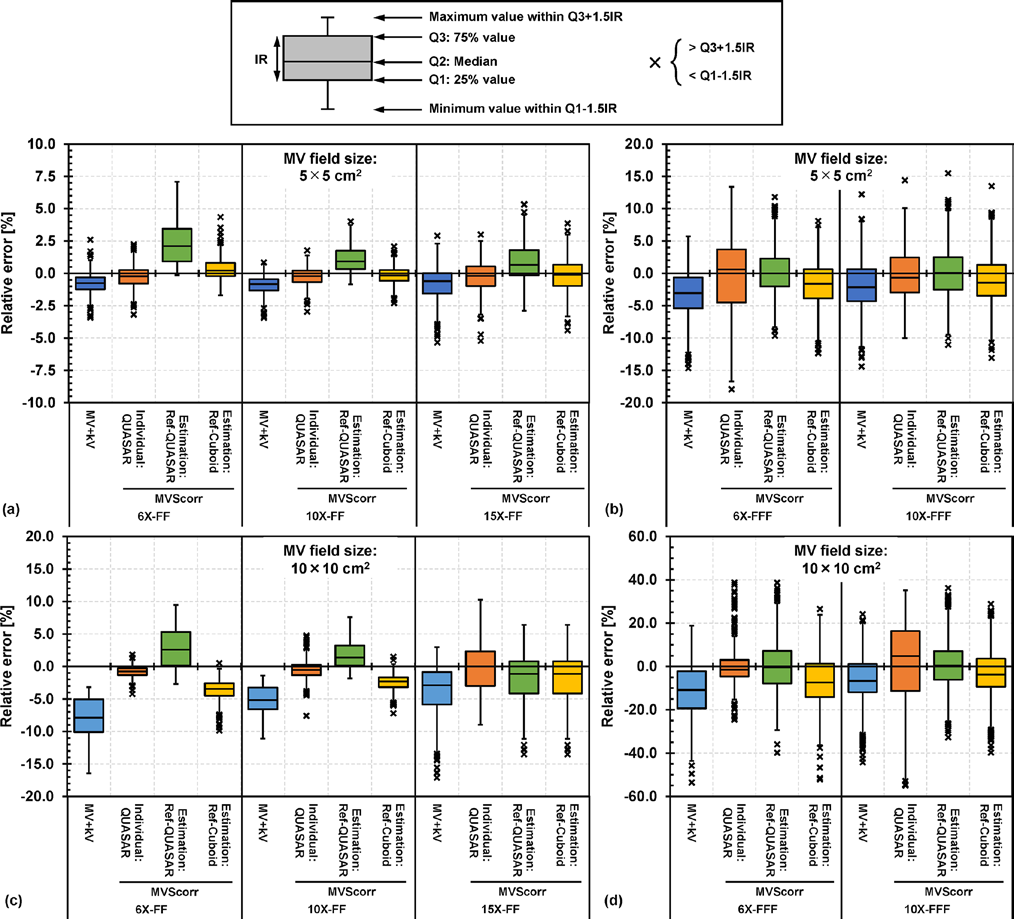
Boxplots of relative errors of image contrasts to reference image for kV image acquired during MV beam irradiation (MV+kV) and MV-scatter corrected (MVScorr) image for (a) FF and (b) FF-free (FFF) beams with FS of 5.0 × 5.0 cm^2^, and for (c) FF and (d) FFF beams with FS of 10.0 × 10.0 cm^2^. The Individual: QUASAR = MV+kV images were corrected by the MV-scatter map acquired using QUASAR at each gantry angle. The Estimation: Ref-QUASAR = MV-scatter maps were estimated from the database and MV-scatter image of the QUASAR phantom acquired by the reference condition (FS: 10.0 × 10.0 cm^2^, dose rate: 400 MU/min, gantry and collimator angles: 0°, and FPD position: 70 cm from isocenter) with 6 MV photon beam with flattening-filter (6X-FF). The Estimation: Ref-Cuboid = MV-scatter maps were estimated from the database and MV-scatter image of the Cuboid phantom acquired by the reference condition with 6X-FF. FF, flattening-filter; FPD, flat panel detector; FS, field size; MV, megavoltage.

Boxplots of the relative errors of the image contrasts to the kV only image for the MV+kV and MVScorr images are presented in Figure 9. The contrasts of the MV+kV images were systematically lower than those of the reference image for all MV beam energies. The image contrast was lower with larger FSs. The median relative error ranges ofthe image contrast for the MV+kV and MVScorr images using “Individual: QUASAR,” “Estimation: Ref-QUASAR,” and “Estimation: Ref-Cuboid” with FSs of 10.0 × 10.0 cm^2^ were −10.9 to −2.9, −1.5 to 4.8, −1.1 to 2.6, and −7.4 to −1.1, respectively. Although the measurement-based method exhibited the greatest improvement in the image contrast, the MV-scatters were corrected even with the estimated $P_{MVSmap}$. As “Estimation: Ref-Cuboid” used $P_{Ref}$ of the Cuboid phantom, the improvement in image contrast was smaller than that of “Estimation: Ref-QUASAR.” The variations in the relative error of the image contrast were large for the FFF beams as the striped band sowing to electric noise were not eliminated by the MV-scatter correction.^11^

## Discussion

Our results demonstrated that the MV-scatter was strongly dependent on the FS, dose rate, and FPD position. In particular, the MV-scatter value factor increased with decreasing MV beam energies under the reference condition for the FF beam. According to the Klein–Nishina formula, which derives the Compton scattering angles, the proportion of side-scattering increases with decreasing MV beam energies.^13^ In our study, the FPD was located perpendicular to the MV beam direction; thus, the proportion of side-scattered X-rays incident on the FPD decreased as the MV beam energy increased. The same trend was observed for the FFF beam. A comparison of the FF and FFF beams indicated that the MV-scatter value factor of the latter was larger under the reference condition. This was because, in the TrueBeam machine, the effective energy of the FFF beam is lower (or the X-ray spectra of the FFF beam is softer) than that of the FF beam, as no beam-hardening effect occurs.^14^

As confirmed by the results of the MV-scatter correction experiment, the MV-scatter was corrected, and the image contrast was improved by both the measurement- and estimation-based correction methods (Figures 8 and 9). The preferred method is the measurement-based one, as supported by Boylan *et al*.^15^ However, in clinical practice, the images for a patient are acquired during the first fraction of each treatment, such that 3D IGRT techniques cannot be applied to the fraction. This is a particularly critical issue for hypofractionated stereotactic ablative radiotherapy, because the number of treatment fractions is small. The estimation-based method can be used in clinical practice. Although $P_{Ref}$ is necessary for using the method, it can be acquired immediately prior to the first fraction. Moreover, a dedicated Monte Carlo (MC) simulation may be an option for estimating the patient-specific $P_{MVSmap}$, which can generate the $P_{MVSmap}$ by inputting planning CT data and the plan information into the MC simulation geometry. The data provided by this study can be used for validating such MC simulation geometry in future work.

Concurrent kV images during MV beam irradiation have been used extensively for real-time 3D IGRT techniques in clinical practice^16^; however, this approach is hindered by the basic and unavoidable problem of MV-scatter. Moreover, FFF beams have been widely applied owing to their shorter irradiation time at a high dose rate. According to our results, the use of an FFF beam will degrade the accuracy of real-time 3D IGRT techniques. Our results indicate that the accuracies of real-time 3D IGRT techniques increase when considering MV-scatter.

One limitation of this study is that it only focused on the MV-scatter map acquired from a kV imaging subsystem perpendicular to the MV beam. Consequently, similar results may be acquired on the Elekta linac as the kV imaging subsystem is perpendicular to the MV beam, but different results may be acquired on the RTRT subsystem because their FPD positions differ. To establish MV-scatter maps for such linacs or subsystems, MV-scatter measurements or MC simulations should be conducted according to the procedure described in this study. In addition, intensity-modulated beams were not considered in this study, which is the other limitation of this study. As supported by Figure 5, MV-scatters were strongly dependent on the field size. Therefore, to apply the MV-scatter correction for the beams, aperture sizes of multileaf collimator on each segment or control point are necessary. Median relative errors of the contrast for MV+kV images acquired by the beams can be estimated by Figure 9.

## Conclusions

To the best of the authors' knowledge, this is the first study to quantify the various dependencies of MV-scatter in detail, including those for FF and FFF beams, on a TrueBeam linac. The MV-scatter was demonstrated to be strongly dependent on the FS, dose rate,and FPD position, and less dependent on the collimator angle. The MV-scatter correction experiment showed that the correction improved the image contrast, even when using the estimation-based correction method. Furthermore, the data generated in this study can be used for validating MC simulations of concurrent MV and kV beam irradiation geometry for patient-specific MV-scatter correction.

## References

[1] De Los Santos J, Popple R, Agazaryan N, Bayouth JE, Bissonnette J-P, Bucci MK, et al Image guided radiation therapy (IGRT) technologies for radiation therapy localization and delivery. Int J Radiat Oncol Biol Phys 2013; 87: 33–45. doi: 10.1016/j.ijrobp.2013.02.021 23664076

[2] Keall PJ, Nguyen DT, O’Brien R, et al Review of real-time 3-dimensional image guided radiation therapy on standard-equipped cancer radiation therapy systems: are we at the tipping point for the era of real-time radiation therapy? Int J Radiat Oncol Biol Phys 2016; 94: 1015–21. 29784460 10.1016/j.ijrobp.2018.04.016 PMC6800174

[3] Shirato H, Shimizu S, Kunieda T, Kitamura K, van Herk M, Kagei K, et al Physical aspects of a real-time tumor-tracking system for gated radiotherapy. Int J Radiat Oncol Biol Phys 2000; 48: 1187–95. doi: 10.1016/S0360-3016(00)00748-3 11072178

[4] Tanabe S, Umetsu O, Sasage T, Utsunomiya S, Kuwabara R, Kuribayashi T, et al Clinical commissioning of a new patient positioning system, SyncTraX FX4, for intracranial stereotactic radiotherapy. J Appl Clin Med Phys 2018; 19: 149–58. doi: 10.1002/acm2.12467 PMC6236847 30273444

[5] Kamino Y, Takayama K, Kokubo M, Narita Y, Hirai E, Kawawda N, et al Development of a four-dimensional image-guided radiotherapy system with a gimbaled X-ray head. Int J Radiat Oncol Biol Phys 2006; 66: 271–8. doi: 10.1016/j.ijrobp.2006.04.044 16820270

[6] Keall PJ, Ng JA, Juneja P, O'Brien RT, Huang C-Y, Colvill E, et al Real-Time 3D image guidance using a standard linac: measured motion, accuracy, and precision of the first prospective clinical trial of kilovoltage intrafraction monitoring-guided gating for prostate cancer radiation therapy. Int J Radiat Oncol Biol Phys 2016; 94: 1015–21. doi: 10.1016/j.ijrobp.2015.10.009 27026307

[7] Hunt MA, Sonnick M, Pham H, Regmi R, Xiong J-ping, Morf D, et al Simultaneous MV-kV imaging for intrafractional motion management during volumetric-modulated Arc therapy delivery. J Appl Clin Med Phys 2016; 17: 473–86. doi: 10.1120/jacmp.v17i2.5836 27074467 PMC4831078

[8] Bertholet J, Toftegaard J, Hansen R, Worm ES, Wan H, Parikh PJ, et al Automatic online and real-time tumour motion monitoring during stereotactic liver treatments on a conventional linac by combined optical and sparse monoscopic imaging with kilovoltage x-rays (COSMIK. Phys Med Biol 2018; 63: 055012. doi: 10.1088/1361-6560/aaae8b 29516868

[9] Nakagawa K, Haga A, Shiraishi K, Yamashita H, Igaki H, Terahara A, et al First clinical cone-beam CT imaging during volumetric modulated Arc therapy. Radiother Oncol 2009; 90: 422–3. doi: 10.1016/j.radonc.2008.11.009 19062117

[10] Iramina H, Nakamura M, Mizowaki T Direct measurement and correction of both megavoltage and kilovoltage scattered x-rays for orthogonal kilovoltage imaging subsystems with dual flat panel detectors. J Appl Clin Med Phys 2020. doi: 10.1002/acm2.12986 PMC7497931 32710529

[11] Luo W, Yoo S, Wu QJ, Wang Z, Yin F-F Analysis of image quality for real-time target tracking using simultaneous kV-MV imaging. Med Phys 2008; 35: 5501–9. doi: 10.1118/1.3002313 19175109

[12] Wallace D, Ng JA, Keall PJ, O'Brien RT, Poulsen PR, Juneja P, et al Determining appropriate imaging parameters for kilovoltage intrafraction monitoring: an experimental phantom study. Phys Med Biol 2015; 60: 4835–47. doi: 10.1088/0031-9155/60/12/4835 26057776

[13] Klein O, Nishina Y Über die Streuung von Strahlung durch freie Elektronen nACh Der neuen relativistischen Quantendynamik von Dirac. Z. Physik 1929; 52(11-12): 853–68. doi: 10.1007/BF01366453

[14] Foster RD, Speiser MP, Solberg TD Commissioning and verification of the collapsed cone convolution superposition algorithm for SBRT delivery using flattening filter-free beams. J Appl Clin Med Phys 2014; 15: 39–49. doi: 10.1120/jacmp.v15i2.4631 PMC5875462 24710452

[15] Boylan CJ, Marchant TE, Stratford J, Malik J, Choudhury A, Shrimali R, et al A megavoltage scatter correction technique for cone-beam CT images acquired during VMAT delivery. Phys Med Biol 2012; 57: 3727–39. doi: 10.1088/0031-9155/57/12/3727 22617805

[16] Keall PJ, Nguyen DT, O'Brien R, Zhang P, Happersett L, Bertholet J, O’Brien R, et al Review of real-time 3-dimensional image guided radiation therapy on standard-equipped cancer radiation therapy systems: are we at the tipping point for the era of real-time radiation therapy? Int J Radiat Oncol Biol Phys 2018; 102: 922–31. doi: 10.1016/j.ijrobp.2018.04.016 29784460 PMC6800174

